# Effects of moxibustion for COVID-19 convalescence: A protocol for systematic review and meta-analysis

**DOI:** 10.1097/MD.0000000000025389

**Published:** 2021-04-09

**Authors:** Yue Zhou, Xiao Yan, Fengjun Ma, Qingchang Xia, Yunping Lu, Wenyuan Li, Shuai Song, Yan Sun, Yuxia Ma, Yuning Ma

**Affiliations:** a Shandong University of Traditional Chinese Medicine; b Affiliated Hospital of Shandong University of Traditional Chinese Medicine, Jinan, Shandong, China.

**Keywords:** COVID-19 convalescence, meta-analysis, moxibustion, protocol, systematic review

## Abstract

**Background:**

Coronavirus disease 2019 (COVID-19) is still spreading around the world. Moxibustion, as a significant therapy in traditional Chinese medicine (TCM), has been widely used to treat COVID-19, especially in recovery period. The study will aim to assess the efficacy and safety of moxibustion for COVID-19 convalescence.

**Methods:**

We will systematically search the relevant randomized controlled trials in the 7 databases from inception to February 2021, including PubMed, MEDLINE, Embase, Cochrane Clinical Trials Database, Web of Science, China National Knowledge Infrastructure and Chinese Biomedical Literature Database. No language and publication status restrictions will be applied. Two reviewers will independently conduct and screen all included studies and the meta-analysis will be performed with RevMan V5.3 (The Cochrane Collaboration, Oxford, England).

**Results:**

The study will provide a high-quality convincing assessment of the efficacy and safety of moxibustion for the treatment of COVID-19 convalescence, which will be published in a peer-reviewed journal.

**Conclusion:**

Our study will give more comprehensive evidence of the effectiveness of moxibustion for COVID-19 convalescence.

**Trial registration number:**

CRD42021230364.

## 1 Introduction

Since the first identification of coronavirus disease 2019 (COVID-19) caused by SARS-CoV-2 in the city of Wuhan, China, in December 2019, COVID-19 has rapidly become a global pandemic, especially among older and vulnerable populations.^[1,2]^ As the pandemic progressed, it has been infecting a large number of people, interrupting routine healthcare services, causing severe disease and associated long-term health sequelae, and resulting in death and excess mortality.^[3–5]^ Currently, plentiful news of unusual public health events possibly due to variants of SARS-CoV-2 have been reported ^[6,7]^ and the global challenge of Covid-19 is still spreading.

It is acknowledged that there is no specific remedy for the treatment of COVID-19 in recovery phase. Given the properties of moxibustion which played a significant role in the treatment and prevention of ancient plague and Severe Acute Respiratory Syndrome (SARS) in 2003,^[8]^ it has been widely used in the treatment and prevention of COVID-19. It is reported that ^[9–11]^ moxibustion therapy could relieve the clinical symptoms such as cough, fatigue, chest distress, poor appetite and so on; reduce the levels of inflammatory indexes, that is, IL-6 and C-reactive protein (CRP); improve the absolute number of peripheral T lymphocyte subsets; as well as alleviate the negative emotions. Numerous studies demonstrated that the clinical therapeutic effect of moxibustion supplemented is significantly better than the simple routine treatment of modern medicine. However, there is no systematic review on the effectiveness and safety of moxibustion in the treatment of COVID-19 in recovery period. Therefore, we will perform a systematic review and meta-analysis of the efficacy of moxibustion in the treatment of COVID-19 convalescence to provide high-quality evidence for clinical application.

## 2 Methods and analysis

### 2.1 Study registration

This systematic review has been registered in PROSPERO (CRD42021230364), which will be conducted in accordance with preferred reporting items for systematic review and meta-analysis protocols 2015 statement.^[12]^

### 2.2 Inclusion criteria for study selection

#### 2.2.1 Types of studies

This review will include clinical randomized controlled trials (RCTs) of COVID-19 convalescence treated with moxibustion. There is no language and publications limitation. Non-RCT, observational study, reviews, experimental study, clinical case reports, and animal research literature will be excluded.

#### 2.2.2 Participants

Patients who were diagnosed with COVID-19 convalescence will be included, regardless of age, gender, educational status or racial restrictions. The diagnosis of COVID-19 includes Chinese or international diagnostic criteria.^[13,14]^

#### 2.2.3 Types of interventions

Patients in the treatment group should be treated with moxibustion alone, or combined with other kinds of therapies, while patients in the control group will receive other treatment without moxibustion.

#### 2.2.4 Outcomes

The primary outcome is the time of disappearance of main symptoms (including short of breath, fatigue, poor appetite, cough disappearance rate, and temperature recovery time), the reexamination of chest X-ray, and the white blood cell count. The secondary outcome will be the associated symptoms (such as dry cough and less phlegm, thirsty upset, chest distress, distention and fullness, loose stool) disappear rate, negative COVID-19 results rate on 2 consecutive occasions (not on the same day), quality of life, occurrence rate of common type to severe form, clinical cure rate, mortality and adverse events.

### 2.3 Search strategy

We will search 7 electronic databases to identify relevant studies from inception to February 31, 2021, which includes PubMed, MEDLINE, Embase, Cochrane Clinical Trials Database, Web of Science, China National Knowledge Infrastructure, and Chinese Biomedical Literature Database. Meanwhile, we will also search Chinese Clinical Trial Registry (ChiCTR) and ClinicalTrials.gov for ongoing trials with unpublished data. The search strategy in PubMed is as follows:

#1 Search: (((((moxibustion [MeSH Terms])) OR (moxa [Title/Abstract])) OR (acupoint [Title/Abstract])) OR (TCM [Title/Abstract])) OR (Traditional Chinese medicine [Title/Abstract]).

#2 Search: ((((((((((COVID-19 [MeSH Terms])) OR (COVID19 [Title/Abstract])) OR (coronavirus disease 2019 [Title/Abstract])) OR (coronavirus disease-19 [Title/Abstract])) OR (SARS-CoV-2 [Title/Abstract])) OR (2019 novel coronavirus disease [Title/Abstract])) OR (2019-nCoV disease [Title/Abstract])) OR (2019 novel coronavirus infection [Title/Abstract])) OR (2019-nCoV infection [Title/Abstract])) OR (2019 coronavirus [Title/Abstract]).

#3 Search: (((((((convalescence [MeSH Terms])) OR (recovery phase [Title/Abstract])) OR (convalescent period [Title/Abstract])) OR (convalescent stage [Title/Abstract])) OR (restoration stage [Title/Abstract])) OR (decubation [Title/Abstract])) OR (recovery period [Title/Abstract]).

#4 Search: (((((((((clinical trials, randomized [MeSH Terms]) OR (randomized controlled trial [MeSH Terms])) OR (controlled clinical trials, randomized [MeSH Terms])) OR (random allocation [MeSH Terms])) OR (allocation, random [MeSH Terms])) OR (controlled clinical trials, randomized [MeSH Terms])) OR (RCT [Title/Abstract])) OR (controlled clinical trial [Title/Abstract])) OR (randomized[Title/Abstract])) OR (trial [Title/Abstract]).

#5 #1 and #2 and #3 and #4.

### 2.4 Data collection and analysis

#### 2.4.1 Selection of studies

**Figure 1:**
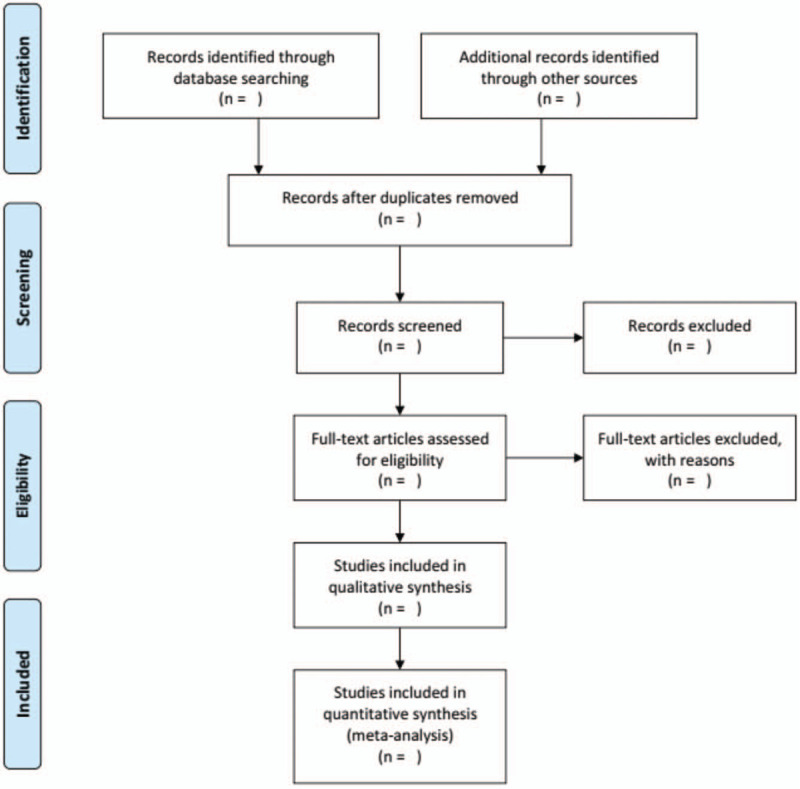
The PRISMA flow diagram.

Two independent reviewers will screen and evaluate the relevant abstracts and titles of all studies based on pre-defined inclusion criteria and then exclude repetitive or ineligible articles with the reasons. The third investigator will solve any disagreement between the 2 reviewers. The process of screening selection is shown in Figure 1.

#### 2.4.2 Data extraction and management

Two reviewers will take charge of data extraction and management according to the retrieval strategy, including the title of the study, the journal, the year of publication, the name of the first author, general information, study design, experimental intervention and intervention time, outcomes, and adverse events. If there is any disagreement between 2 reviewers in the data extraction, the group will arbitrate and make decisions together.

#### 2.4.3 Dealing with missing data

When it comes to missing or unclear data, we will try our best to contact the corresponding author for more detailed information. If it fails, we will analyze it based on available data.

#### 2.4.4 Assessment of risk of bias

The Cochrane Handbook for systematic reviews of interventions Version 6 will be performed to assess a broad category of biases in the included studies. We will evaluate biases from the following 7 aspects: random sequence generation, allocation concealment, blinding of the participants and personnel, blinding of the outcome assessments, incomplete outcome data, selective reporting, and other sources of bias. These studies will be assigned as low risk, high risk or unclear risk. Inconsistencies will be resolved by discussion with other reviewers.

#### 2.4.5 Assessment of quality of evidence

The Grading of Recommendations Assessment, Development and Evaluation system will be used to judge the overall quality of evidence supporting outcomes in this work. And the quality of evidence will be defined as high, moderate, low, or very low.

#### 2.4.6 Measures of treatment effect

In the study, Risk ratios with 95% confidence intervals (CIs) for analysis will be presented for dichotomous data while standard mean difference (SMD) or mean difference (MD) with 95% confidence intervals will be used for analyzing the continuous data.

#### 2.4.7 Assessment of heterogeneity

Cochrane X^2^ and *I*^2^ tests will be used for the evaluation of heterogeneity. It is acknowledged that if *P* ≥ .05 and *I*^2^ ≤ 50%, the assessment of heterogeneity can be neglected; and there is great heterogeneity between included studies if *P* < .05 and *I*^2^ > 50%.

#### 2.4.8 Assessment of reporting bias

If there are over 10 studies included in the meta-analysis, funnel plots will be used to detect the reporting biases.^[15]^

#### 2.4.9 Data synthesis

We will take advantage of Review manager software (RevMan) V.5.3 for data analysis and synthesis. Data will be processed with a fixed-effect model if no statistical heterogeneity was observed among the results (*P* ≥ .05 and *I*^2^ ≤ 50%). Meanwhile, the random-effect model will be put into use, if *P* < .05 and *I*^2^ > 50%.

#### 2.4.10 Subgroup analysis

If available, we will conduct a subgroup analysis based on different interventions, controls, durations of treatment, and outcome measures.

#### 2.4.11 Sensitivity analysis

Sensitivity analyses will be carried out to investigate the robustness of the study conclusions. Methodological quality, sample size, and the effect of missing data will be included. Therefore, the impact of low-quality studies on the overall results will be evaluated.

#### 2.4.12 Ethics and dissemination

This work does not require relevant ethical review because there is no data linked to individual patient or animal information. Our research results will be shared and demonstrated through peer-reviewed journals.

## 3 Discussion

The rapid spread of coronavirus disease 2019 caused by SARS-CoV-2 has a major impact on human health globally.^[16]^ In recovery period, some patients’ results of viral nucleic acid test have turned negative, but the patients still have fatigue, cough, poor mental state, et al. Moxibustion has a long history and plays an crucial role in the treatment of diseases because of its vital roles in regulating immune system and inhibiting cytokine storm. Thus, it has been widely reported in prevention and treatment of COVID-19. Nevertheless, no systematic review relevant to moxibustion for COVID-19 convalescence has been published. In this work, we will systematically review to validate the effectiveness of moxibustion in COVID-19 recovery period. We hope that the results of this review will give more appropriate evidence-based decisions to assist clinicians during the decision-making process when dealing with COVID-19 convalescence.

## Author contributions

**Data curation:** Yue Zhou.

**Formal analysis:** Xiao Yan, Fengjun Ma.

**Methodology:** Yue Zhou, Qingchang Xia, Wenyuan Li, Shuai Song.

**Project administration:** Yuxia Ma.

**Resources:** Yue Zhou, Yan Sun.

**Software:** Yue Zhou, Fengjun Ma, Yunping Lu.

**Visualization:** Xiao Yan.

**Writing – original draft:** Yue Zhou, Yuxia Ma.

**Writing – review & editing:** Yuning Ma.
